# Development of a cartilage composite utilizing porous tantalum, fibrin, and rabbit chondrocytes for treatment of cartilage defect

**DOI:** 10.1186/s13018-015-0166-z

**Published:** 2015-02-07

**Authors:** Kamal Jamil, Kien-Hui Chua, Samad Joudi, Sook-Luan Ng, Nor Hamdan Yahaya

**Affiliations:** Department of Orthopaedic and Traumatology, Faculty of Medicine, Universiti Kebangsaan Malaysia, Jalan Yaacob Latiff, Bandar Tun Razak, 56000 Cheras, Kuala Lumpur Malaysia; Department of Physiology, Faculty of Medicine, Universiti Kebangsaan Malaysia, Jalan Raja Muda Abdul Aziz, 50300 Kuala Lumpur, Malaysia

**Keywords:** Cartilage composite, Porous tantalum, Chondrocyte proliferation, Cartilage defect, Fibrin

## Abstract

**Objective:**

Functional tissue engineering has emerged as a potential means for treatment of cartilage defect. Development of a stable cartilage composite is considered to be a good option. The aim of the study was to observe whether the incorporation of cultured chondrocytes on porous tantalum utilizing fibrin as a cell carrier would promote cartilage tissue formation.

**Methods:**

Rabbit articular chondrocytes were cultured and seeded onto tantalum with fibrin as temporary matrix in a composite, which was divided into three groups. The first group was kept *in vitro* while a total of 12 constructs were implanted into the dorsum of mice for the second and third groups. The implanted tissues were harvested after 4 weeks (second group) and after 8 weeks (third group). Specific characteristic of cartilage growth were studied by histological and biochemical assessment, immunohistochemistry, and quantitative PCR analysis.

**Results:**

Histological and biochemical evaluation of the formed cartilage using hematoxylin and eosin and Alcian blue staining showed lacunae chondrocytes embedded in the proteoglycan rich matrix. Dimethylmethylene blue assay demonstrated high glycosaminoglycans content in the removed tissue following 8 weeks of implantation. Immunohistochemistry results showed the composites after implantation expressed high collagen type II. Quantitative PCR results confirmed a significant increase in cartilage associated genes expression (collagen type II, AggC, Sox 9) after implantation.

**Conclusion:**

Tantalum scaffold with fibrin as cell carrier promotes chondrocyte proliferation and cartilaginous tissue formation. Producing hyaline cartilage within a stable construct of tantalum and fibrin has a potential for treatment of cartilage defect.

## Introduction

Around two million individuals are affected worldwide with arthritis [1]. These individuals require hospitalization, which is a matter of concern. Total joint replacement is the usual end-stage treatment, but implant longevity is an issue as more than half of the affected individuals is below 65 years old [2-7]. Damage to articular cartilage leads to premature arthritis. This chondral lesion was reported to be present in 60% of all patients aged between 40–50 years during arthroscopy [1,7,8]. Due to its avascular nature and ineffective response to injury, articular cartilage has limited potential to heal, even so in larger defects. Partial thickness injuries do not heal and merely stimulate minimal reaction to adjacent chondrocytes in the form of cell replication and matrix turnover, whereas full thickness injuries penetrating subchondral bone produce normal healing response but eventually fills the defect with fibrocartilage [9]. Fibrocartilage resists to tension, in contrast to normal hyaline cartilage which resists compressive forces. Therefore, there is an urgent need for a method for effective biological healing and regeneration of cartilage to prevent premature arthritis.

There are various methods of operative treatment employed to treat osteochondral injury. Marrow stimulation and resurfacing techniques have been advocated but they have their limitations while dealing with large defects and producing hyaline cartilage [9-15]. Autologous chondrocyte implantation (ACI) which was brought into attention by Brittberg et al. in 1994 is quite promising but this technique is expensive and highly technically dependent [10]. It requires two surgeries and needs laboratory support to grow the cells [13].

Functional tissue engineering is a novel approach to enhance tissue regeneration and provides the possibility of producing tissue that is biomechanically, biochemically, and histomorphologically similar to the native tissues [7]. Scientists and engineers working in this promising field are taking steps to make those ideas a reality, working to supply biological substitutes or living tissue. A previous study by Munirah et al. for articular cartilage restoration focused on engineering autologous cartilage construct using human and ovine chondrocytes incorporated with the autologous fibrin as biomaterial [16]. The pre-clinical study, conducted in sheep, was designed to evaluate the performance of the autologous ‘chondrocytes-fibrin’ construct implantation in a simulated clinical application prior to undertaking the definitive pre-clinical and clinical investigations. However, it has its own limitation. Resulted ‘chondrocytes-fibrin’ constructs were too soft to hold into the defect independently. Technically, periosteal patch were used to secure the implant into the defect.

There is continuous quest for the development of a new and more reliable technique to restore or reconstruct osteochondral defects. The development of a composite with the biochemical and mechanical properties of an osteochondral graft with better integration properties and without the need for autologous osteochondral graft harvesting or allogeneic tissue would be attractive. Tantalum, an elemental metal which is strong, biocompatible, and corrosive-resistant, has been widely used in the field of orthopedics due to its low modulus of elasticity and low frictional characteristics. It also has high, interconnected porosity with excellent bone in growth properties. In the present study, we incorporated cultured chondrocytes on porous tantalum utilizing fibrin as a cell carrier to support *in vitro* chondrogenesis. Following this, we compared it with constructs implanted for 4 and 8 weeks, which hypothetically has superior cartilaginous development.

## Materials and methods

The study was conducted after receiving the ethical approval from the animal and research ethics committee of our university. The project approval number from Universiti Kebangsaan Malaysia was FF-307-2009.

### Tantalum preparation

**Figure 1:**
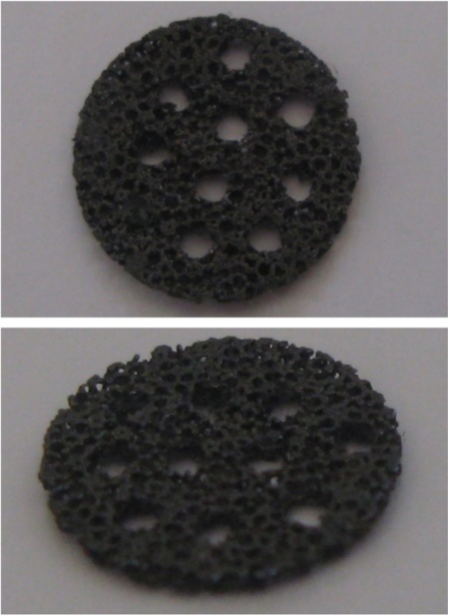
**Tantalum scaffold in 10 mm in diameter and 0.5 mm in thickness.**

All scaffolds were prepared from 2 cm in diameter and 10 cm length of Zimmer tantalum screw using Machine Wire Cut (Hitachi, H-Cut 203 M20) in thickness of 0.5 mm and Milling Machine Cut to make holes Ø1.0 on the both surface of tantalum (Figure 1).

### Chondrocyte cell preparation

Six New Zealand white rabbits age 2 months old and weighting 2.0–2.3 kilograms were used as experimental animals. Blood was taken to get autologous plasma and serum before the animal was sacrificed with overdosage of phenobarbital. Full-thickness cartilage was harvested from the knee, hip joints, and patella articular surface under aseptic technique. Each cartilage was separated from the perichondrium and subchondrium bone, minced into small pieces (1 mm^3^), washed with Dulbecco phosphate buffer saline (DPBS; pH 7.2; Gibco, Grand Island, NY, USA) containing 100 μ/ml penicillin and 100 μ/ml streptomycin (PBS, Gibco). Finally, the samples were washed with DPBS one more time. Cartilages were digested using 0.3% collagenase type II (Gibco) in a shaker incubator (Stuart Scientific, Redhill, UK) at 37°C for 90 min. Samples were centrifuged (500 × *g*) for 10 min to get the cell pellet. The supernatant was removed and the cell pellet was washed with 15 ml DPBS to remove the remaining enzyme. The sample was centrifuged for 10 min to get the final pellet cells. Isolated chondrocytes were seeded in six well plates containing the culture medium (Ham’s F12 and Dulbecco’s modified medium 1:1 + 1% ascorbic acid + 10% autologous serum + antibiotic/antimycotic + 1% glutamine). All cultures were maintained in 5% CO_2_ incubator (Jouan, Duguay, Trouin, SH) at 37°C with every 3 days medium change. Once confluence, the primary cultures were trypsinized using trypsin-EDTA 0.125% (Gibco). The cells pellet were then cultured in large-scale 175 cm^2^ culture flask (Falkon, Franklin Lake, NJ, USA) at a density of 5,000 cells/cm^2^. Chondrocyte morphologic features were examined every day using inverted light microscope (Olympus, Shinjukuku, Tokyo). Upon confluence, the cells were trypsinized and mix with plasma before put on to the tantalum. Fibrin polymerization took place after CaCl_2_ solution was added into the mixture. The cell-fibrin-tantalum constructs were maintained for 3 days in the culture before implantation. It was cultured in the same medium for cell expansion, in the six-well dishes and maintained in the CO_2_ incubator.

### Construct implantation

**Figure 2:**
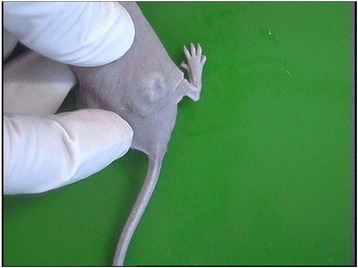
**Construct implanted over dorsum of mice.**

Under aseptic technique, surgery was performed under sedation. Cocktail drug which consist of ketamine, xylazine, and zoletil was given according to body weight intramuscular. Surgical incision was made at the dorsum of nude mice, and two constructs were implanted on the left and right side of the dorsum (Figure 2). The skin was then sutured using 6/0 vicryl (12 complexes totally at six mice). Care of the nude mice was carried out following the animal facility guideline of the Animal Unit, UKM.

### Harvest of composite

Three mice were sacrificed after 4 weeks and another three mice were sacrificed after 8 weeks to remove the constructs.

### Histological and biochemical assessment

The obtained cartilage underwent histology and biochemical evaluation. Constructs were fixed in 10% formalin for 24 h at 4°C and processed using standard histological technique which finally embedded in paraffin. The tissues were cross sectioned (5 μm thick) and stained with hematoxylin and eosin, and Alcian blue according to standard procedures. For biochemical assessment, the amount of glucosaminoglycans (GAGs) was measured using the dimethylmethylene blue (DMMB) assay (12) and the total amount of GAGs per dry weight of formed cartilage (μg/ml/mg) was recorded.

### GAG quantification

For quantitative measuring of GAGs, the total GAGs content of cell/polymer constructs was determined using papain digestion and the dimethylmethylene blue dye method. Samples were lyophilized for 24 h and then digested under sterile conditions with papain type III (Sigma) at 125 mg/ml in a buffer of 0.1 M NaH_2_PO_4_, 5 mM methylenediaminetetraacetic acid, and 5 mM cysteine hydrochloride at pH 7.0 overnight at 60°C before the dye was added. Dimethylmethylene blue stock solution was made using dimethylmethylene blue, sodium chloride, glycine, sodium aziade in 1 N hydrochloride, and water. A spectrophotometer set at a wavelength of 520 nm was used to measure the optical density of the digested samples. Glycosaminoglycan was measured and reported in micrograms per milliliter per milligram of dry weight tissue.

### Immunohistochemistry

Formalin-fixed tissues were sectioned and treated with proteinase K at 37°C for 60 min before washed three times with tris buffered saline (TBS, DAKO Cytomation). The sections were then treated with peroxidase block at 37°C for 10 min prior to incubation with antibody. Two antibodies were used, anti-type I and anti-type II collagen were diluted 1:150 with diluent (DAKO Cytomation) and applied to different sections for 40 min at 37°C. After washing with TBS, the sections were reacted with horseradish peroxidase (HRP) for 40 min at 37°C. After washing again with TBS, the signal was finally visualized as a brown reaction product from the peroxidase substrate 3,3′-diaminobenzidine (DAB).

### Quantitative gene expression by real-time PCR

Total RNA was extracted from removed tissue engineered using TRI reagent (Molecular Research Center, Cincinnati) according to the manufacturer. Cartilage differentiation (type II collagen, SOX9 transcription factor, and aggrecan core protein), dedifferentiation (type I collagen), and hypertrophy (type X collagen) gene expression level was quantified by real-time PCR technique. Primer sequence used was designed with Primer 3 software based on the GeneBank database sequences corresponding to the specific gene Accession Number. The reaction kinetic and specificity of each primer set was verified with standard curve and melting profile. The quantitative RT-PCR protocol was performed in a Bio-Rad iCycler with profile of cDNA synthesis for 30 min at 50°C, pre-denaturation for 2 min at 94°C and PCR amplification for 38 cycles with 10 s at 94°C, 10 s at 60°C, and 30 s at 72°C. This series of cycles was followed by melt curve analysis to check the reaction specificity. The data was analyzed using Bio-Rad iCycler software. The expression level of each targeted gene was normalized to the house keeping gene - GAPDH.

### Statistical analysis

Data for GAGs amount in each construct at all three environments (*in vitro*, *in vivo*, 4 and 8 weeks) were collected from 16 samples. Values were presented as mean ± standard error of mean (SEM). ANOVA and Student’s *t* test were used to compare data between groups. Differences were considered significant when *p* < 0.05. Data collected from quantitative parameter was analyzed using independent *t* test or Mann–Whitney test. Values were presented as mean ± SEM. Differences at 5% level were considered significant. All analyses were performed using SPSS 10.0 software.

## Results

### Cellular morphology and construct gross appearance

**Figure 3:**
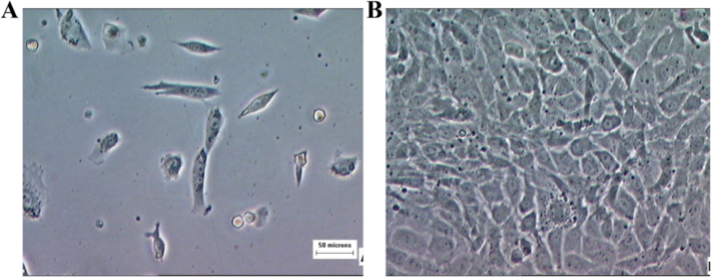
**Chondrocytes morphology at primary culture. (A)** Small, polygonal shape at early stage. **(B)** Chondrocytes reached confluency after 1 week in culture. Magnification × 200.

**Figure 4:**
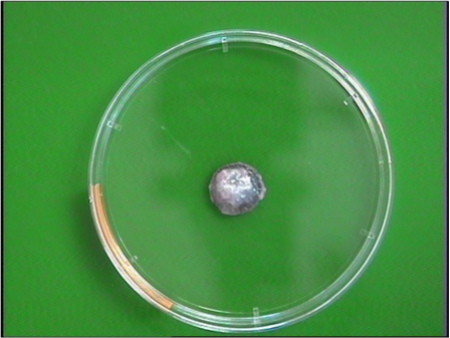
**Construct of tantalum-chondrocyte-fibrin after implantation.**

In monolayer culture, chondrocytes exhibited small and polygonal shape and they continued to proliferate and reached confluency after 1 week (Figure 3). After construct implantation, all nude mice survived without any clinical signs of morbidity or mortality. Grossly, the constructs demonstrated stable form of implant with no signs of tissue reaction. After 4 and 8 weeks implantation, three nude mice were sacrificed at 4 weeks and another three nude mice at 8 weeks to harvest the constructs. Tissue-engineered cartilage appeared white, smooth, glistening, and felt to be firm, resisting to compression resembling a normal hyaline cartilage (Figure 4).

### Histological results

**Figure 5:**
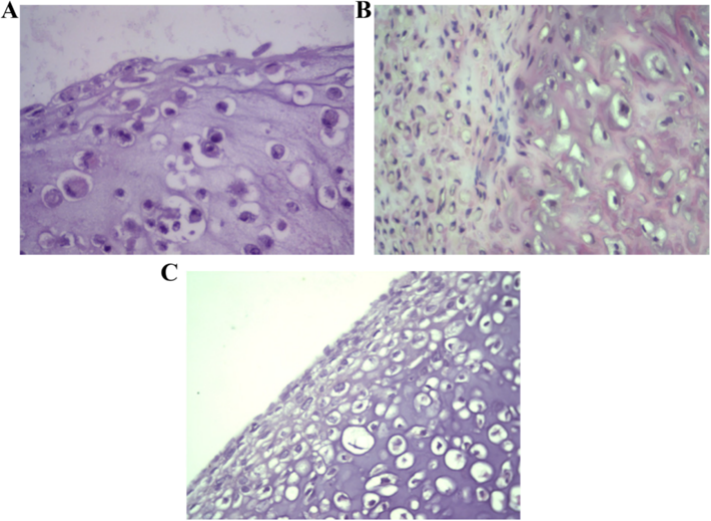
**H&E staining of tissue in** ***in vitro*** **(A), 4 weeks** ***in vivo*** **(B), and 8 weeks** ***in vivo*** **(C)** ***.*** *In vitro* construct showed immature cells with scanty basophilic background, while both *in vivo* constructs demonstrated evenly distributed lacunae cells within basophilic matrix. However, 8-week construct exhibited more mature cells throughout extracellular matrix.

The histological results at day 1 *in vitro* and 4 weeks and 8 weeks *in vivo* constructs were variable. *In vitro* samples demonstrated immature lacunae cells with scanty basophilic matrix background. *In vivo* samples at 4 weeks implantation had more evenly distributed lacunae cells, but immature with reactive nucleus in the tissue. Samples at 8 weeks implantation revealed evenly distributed lacunae embedded within a basophilic matrix (Figure 5). At both 4 and 8 weeks, the tantalum remains intact and incorporated as a composite with the cells and fibrin.

**Figure 6:**
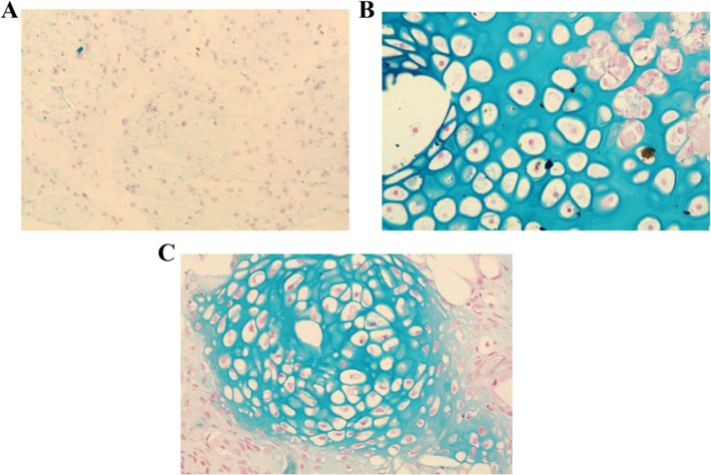
**Alcian blue staining for proteoglycan of tissue** ***in vitro*** **(A), 4 weeks** ***in vivo*** **(B), and 8 weeks** ***in vivo*** **(C).** Formed tissue obtained from 8 weeks *in vivo* construct revealed abundant cells in clusters with positive staining throughout the extracellular matrix compared to 4 weeks *in vivo* and *in vitro* construct.

Alcian blue staining as specific staining for proteoglycan showed immature cells with scanty hyalinized matrix background in *in vitro* samples. Following 4 weeks implantation, the cells appeared rounded in clusters with extracellular matrix-stained blue, indicating production of proteoglycan. *In vivo* samples at 8 weeks demonstrated abundant mature cells in clusters, with extensive staining throughout the extracellular matrix (Figure 6).

### GAG quantification of constructs

**Figure 7:**
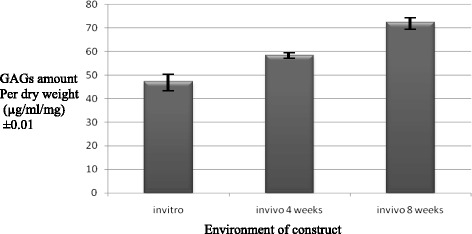
**Comparison of mean of GAGs amount in various environments (μg/ml/mg) in increasing pattern from** ***in vitro*** **to** ***in vivo*** **construct at 4 and 8 weeks.** (*p* < 0.05).

A mean amount of GAGs per dry weight of formed tissue in *in vitro*, 4 weeks *in vivo*, and 8 weeks *in vivo* was 47.32 ± 3.12, 58.37 ± 1.28, and 72.40 ± 2.72, respectively (Figure 7). Statistical analysis using ANOVA method showed increasing amount of GAGs between groups from *in vitro* (day 1) to *in vivo* (4 and 8 weeks) which is significant (*p* < 0.005). This demonstrated high-quality cartilage tissue formed with increasing implantation period in the *in vivo* environment.

Quantitative comparison between *in vitro* and 4 weeks *in vivo* construct using Student *t* test demonstrated significant difference between the total amount of produced GAGs (*p* = 0.013). Comparison of total amount of GAGs per dry weight between 4 and 8 weeks *in vivo* demonstrated significant difference as well (*p* = 0.006). Samples at 8 weeks implantation formed more high-quality cartilage with higher amount of GAGs production.

### Immunohistochemistry of constructs

**Figure 8:**
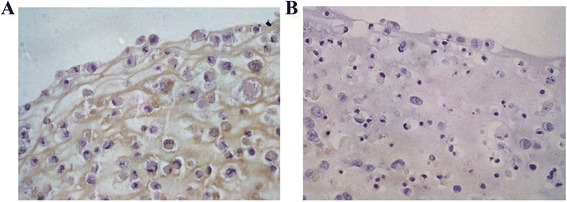
**Immunohistochemical staining for** ***in vitro*** **construct shows brownish deposition for localization of collagen type I (A) but no expression for collagen type II (B).**

Following implantation, tissue-engineered cartilage showed progression from immature tissues towards maturity by 4 and 8 weeks. By 8 weeks, the histological feature exhibited lacunar being formed with increase in extracellular matrix. *In vitro* construct exhibited poorly distributed cartilage cells and less lacunar formed. We observed moderate expression towards collagen type I around the underdeveloped pericellular matrix but no expression for collagen type II (Figure 8).

**Figure 9:**
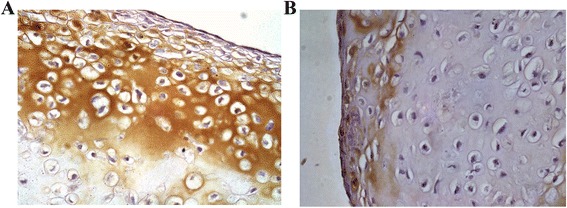
**Immunohistochemical staining of** ***in vivo*** **construct shows strong reaction towards collagen type II (A) but weak reaction with collagen type I with brown discoloration extracellularly (B).**

*In vivo* construct showed strong expression towards collagen type II with brown discoloration pericellular and throughout the matrix. Weak immunopositivity was observed towards collagen type I (Figure 9).

### Real-time PCR analysis on the constructs

**Figure 10:**
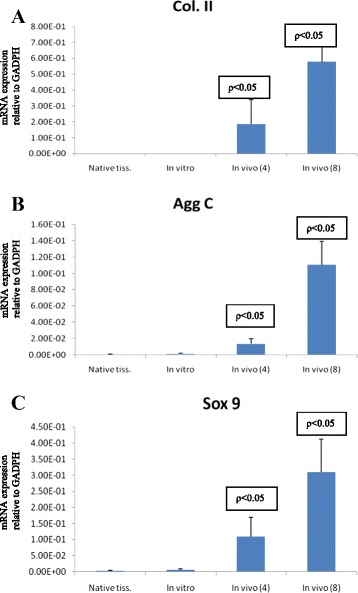
**Comparison between** ***in vivo*** **constructs (4 and 8 weeks) to** ***in vitro*** **for the quantitative RT-PCR analysis (** ***p*** **< 0.05).** It determined the gene expression of collagen type II **(A)**, aggrecan **(B),** and Sox 9 **(C)**. *Native* isolated RNA from normal native cartilage.

Cartilage-associated genes (collagen type II, aggrecan core protein, Sox 9 transcription factor) showed significantly higher expression in *in vivo* constructs at 4 and 8 weeks compared to *in vitro* construct. This proved the greater ability of *in vivo* tissue-engineered cartilage to produce mature cartilage phenotype. However, we observed no significant difference between *in vivo* constructs at 4 and 8 weeks, except for aggrecan core protein (Figure 10).

**Figure 11:**
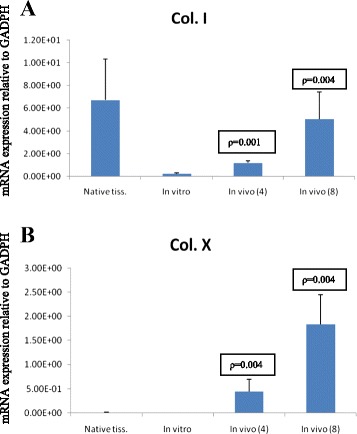
**Comparison between in vivo constructs (4 and 8 weeks) to** ***in vitro*** **for the quantitative RT-PCR analysis (** ***p*** **< 0.05) of gene expression of collagen type I (A) and collagen type X (B).** *Native* isolated RNA from normal native cartilage.

Fibrocartilage (collagen type I) and hypertrophic (collagen type X) markers showed moderately high gene expression levels. There was significant difference between *in vitro* and *in vivo* constructs at 4 and 8 weeks but no difference was observed between *in vivo* constructs at the same period (Figure 11).

## Discussion

Areas of research regarding cartilage repair have essentially focused on cultured cells supported in the engineered tissue. The delivery of this tissue is one of the challenges which include developing an appropriate scaffold and adhesive to provide matrix for the cells to grow. The term ‘chondroconductive’ was described by Gordon et al. and defined as providing a scaffold for the growth of cartilage and supporting structures [17]. They found that porous tantalum is chondroconductive *in vitro* in dynamic environment. The potential of porous tantalum has been further evaluated by Mardones et al. in the development of cartilage-tantalum composite [18]. In this study, periosteum from rabbits were placed on top of porous tantalum cylinders and cultured under chondrogenic conditions for 6 weeks. The findings show hyaline-like cartilage on the surface of the cylinders while the pores of the scaffold were filled with fibrous fixation. Mechanical testing was also performed which showed properties similar to normal rabbit cartilage. This cartilage from femoral condyles were made into osteochondral plugs and tested against the composite. It showed a stress–strain curve with characteristics typical of normal cartilage responding to a load. These findings have been the basis of further studies to consider cartilage-tantalum composite as an option for resurfacing and arthroplasty procedures. The abovementioned technique has limitation in term of making different thickness of cartilage from periosteum tissue. In the present study, we used fibrin to bind cultured chondrocytes and seeded onto tantalum in order to increase the feasibility of making various thickness of cartilage tissue.

The limitation of the study was the relatively short study period. An animal study by Shao et al. using allogeneic bone marrow mesenchymal stem cells (BMSC) seeded onto fibrin glue matrix and medical-grade polycaprolatone (mPCL) revealed deterioration of the transplant after 6 months, despite early good results [19]. Admittedly, if the present study could have been conducted over the same time period with the previous reported study, we could have compared it better. Furthermore, the subcutaneous environment of our construct on the dorsum of nude mice might not be representative of a true clinical situation where intraarticular environment is involved. Mrosek et al. reported an in vivo study where cylindrical osteochondral defects were created on the medial and lateral condyles of ten rabbits and filled with tantalum/periosteum or poly-epsilon-caprolactone/periosteum biosynthetic composites [20]. Even though subchondral bone regeneration was excellent, neo-cartilage formation from periosteum supported by a scaffold was inconsistent [20]. However, Munirah et al. proved that autologous chondrocyte-fibrin construct (ACFC) promotes early chondrogenesis by inducing hyaline-like cartilage regeneration at 12 weeks post-surgery in a sheep model [16]. Biomechanical testing is also appropriate as the next step for tantalum-chondrocyte-fibrin composite.

In the present study, the tissue-engineered cartilage showed morphological features of polygonal that became spindle-shaped and elongated during monolayer culture. These findings are similar to previous studies, which portrays the dedifferentiation in culture as the cultured chondrocytes lost their phenotype to adopt fibroblastic traits [15,21,22]. Our 2-month results also demonstrated that cultured chondrocytes within the fibrin and tantalum scaffold are able to differentiate and produce hyaline-like cartilage tissue. Histological evaluation of *in vivo* samples revealed chondroblast and chondrocytes surrounded by matrix containing hyaline. Seeded cell on tantalum *in vitro* was uniform and homogenous and contained immature chondrocytes with low concentration of GAGs in the hyalinized matrix, while in implanted constructs, the total amount of GAGs per dry weight of tissue significantly increased. This was further proven by strong immunopositivity by immunohistochemistry at 4 and 8 weeks, respectively. It also means that by 2 months, the cartilage has not featured any age-related changes yet. Studies with longer duration need to be done to evaluate the integrity of the cartilage. We also found that cartilage-associated genes (collagen type II, AggC, Sox 9) showed high gene expression after 8 weeks. This supports our hypothesis and suggested that the cartilage composite had produced a good matrix for the chondrocytes to differentiate. Similar findings were reported by previous studies of human articular chondrocytes [15,23]. Fibrocartilage and hypertrophic markers even though present showed much lower expression level. Sasano et al. also discovered that chondrocytes synthesize collagen type I and accumulate the protein in the matrix during his study of rat tibial articular cartilage [15,24].

These findings are in accordance with previous studies in proving that autologous chondrocyte and fibrin composite produced good-quality cartilage-like tissue [21,25-32]. In addition, we overcome the concern of fibrin glue detachment and degradation by providing a good scaffold with tantalum. Tantalum usage has been well established in clinical settings due to its biocompatibility and highly interconnected porosity which permits physiologic bone ingrowth and healing [17,33-41]. By 8 weeks, this tantalum-chondrocyte-fibrin composite show good promise in providing solution for cartilage defect. Clinically, this cartilage construct may be implanted arthroscopically and remain *in situ* with a stable scaffold. This advantage offers the option of a single surgery without the use of a periosteal patch which can be unstable.

## Conclusion

We showed that tantalum scaffold with fibrin as cell carrier promotes cellular proliferation and cartilaginous tissue ormation of rabbit articular chondrocytes. Engineered cartilage resulted from *in vivo*-implanted construct demonstrated high-quality hyaline-like tissue by histological and biochemical assessment, GAG quantification, immunohistochemistry, and real-time PCR. This early results highlight the potential of tantalum-chondrocyte-fibrin composite in treatment of cartilage defect.
